# Abdominal Wall Endometriosis: Two Case Reports and Literature Review

**DOI:** 10.3390/medicina56120727

**Published:** 2020-12-21

**Authors:** Bogdan Doroftei, Theodora Armeanu, Radu Maftei, Ovidiu-Dumitru Ilie, Ana-Maria Dabuleanu, Constantin Condac

**Affiliations:** 1 Faculty of Medicine, University of Medicine and Pharmacy “Grigore T. Popa”, University Street, no 16, 700115 Iasi, Romania; bogdandoroftei@gmail.com (B.D.); dr.radu.maftei@gmail.com (R.M.); dabu_93@yahoo.com (A.-M.D.); 2 Clinical Hospital of Obstetrics and Gynecology “Cuza Voda”, Cuza Voda Street, no 34, 700038 Iasi, Romania; 3 Origyn Fertility Center, Palace Street, no 3C, 700032 Iasi, Romania; 4 Department of Biology, Faculty of Biology, “Alexandru Ioan Cuza” University, Carol I Avenue, no 20A, 700505 Iasi, Romania; 5 Faculty of Medicine, “Lucian Blaga” University, Victoriei Avenue, no 10, 550024 Sibiu, Romania; costicondac@gmail.com

**Keywords:** endometriosis, scar endometriosis, ultrasound, genital microbiota

## Abstract

*Background and objectives:* Abdominal wall endometriosis, also known as scar endometriosis, is a rare condition that is becoming increasingly common. The recent rise in incidence is attributed primarily to the surge of cesarean births, figures that could be influenced in a positive manner considering the improvements brought towards the ultrasound diagnostic methods that have been made in recent years. *Materials and Methods:* Here we report the cases of two Caucasian women of 38- (G2P2) and 36-years old (G1P1), both subjected to an ultrasound examination due to a specific symptomatic panel reported during anamnesis. Independently of the current status, in the first patient, there were no reported symptom-specific associations with endometriosis, but she had a known history of mild hereditary thrombophilia; the second woman suffered from two conditions positively associated with endometriosis. *Results:* In both cases, abnormal structures were revealed, with the diagnostic(s) of endometriosis being histologically confirmed based on a set of features observed during the investigation. *Conclusions:* This paper aims to highlight the importance of reducing cesarean births and to consider the diagnosis of scar endometriosis in women with a history of obstetric or gynaecological surgeries who present with cyclic, recurrent abdominal pain.

## 1. Introduction

Endometriosis is defined as the presence of functional ectopic uterine tissue glands and stroma outside of the uterine cavity [1]. The aetiology of endometriosis is complex and multifactorial, and the theories describing its pathogenesis are yet to be fully confirmed [2,3,4,5]. Although a benign condition, endometriosis is a highly debilitating disease and is further aggravated by its association with dysmenorrhoea, dyspareunia, menstrual irregularities, and infertility, with significant detrimental effects on social, occupational, and psychological functioning [6].

In most cases, endometriosis is located within the pelvis and mainly affects women of reproductive age. However, endometrial implants can be found outside the pelvis and can affect different organs, causing a variety of symptoms with a cyclical pattern of manifestation. The main sites targeted by extra pelvic endometriosis include the bowel, bladder, ureter, kidney, lymph nodes, lungs, and pleura, and also the abdominal wall [4].

Scar endometriosis is a rare form of endometriosis that typically occurs after gynecological or obstetric procedures such as caesarean sections, various laparoscopic procedures that require uterine cavity manipulation, needle tract amniocenteses, or perineal episiotomies [7,8,9].

Regarding the pathogenesis of scar endometriosis, the most widely accepted hypothesis is that the endometrial cells are directly implanted via an iatrogenic process. Other theories have been proposed, such as lymphatic or hematogenous dissemination, metaplastic transformation, and cell immunity modification [10].

Scar endometriosis is a challenging diagnosis to establish because it is reached rather by exclusion than as a positive diagnosis process. This is due to the non-specificity of symptoms such as cyclic abdominal pain, swelling, or bruising of the scar post-surgery. The ultrasound may be useful for preoperative diagnosis and evaluation of invasion into the subjacent anatomical planes. Preoperative evaluation of invasion is very important because, in case of large muscle invasion or deep invasion into the omentum or bowel, the participation of a general surgeon is necessary, with all the logistical implications [4].

Herein, we present the cases of two patients suspected of suffering from abdominal wall scar endometriosis following caesarean births. Both patients were systematically evaluated preoperatively with 2D and Doppler ultrasound, the endometriosis being later on confirmed microscopically after surgical excision.

## 2. Patients

### 2.1. Case 1

A 38-year-old Caucasian woman with a history of two caesarean sections (G2P2), the last of which was in November 2015), presented to our outpatient department with complaints of a painful, palpable, small, firm mass of approximately 2 to 3 cm, located in the lower abdomen wall, at the site of the caesarean section scar, with no mobility to the deeper anatomical planes. The patient described her pain as relatively cyclic and timed the onset of the symptoms at 3 months after her last C-section. The patient was known to suffer from a mild form of hereditary thrombophilia, which required prophylaxis during her two pregnancies and had no other known endometriosis lesions elsewhere. The rest of the patient’s history was unremarkable: no natural births and no complications at the time of the two C-sections, no other relevant family history, no smoking or alcohol consumption, and no medications at the time of presenting.

**Figure 1:**
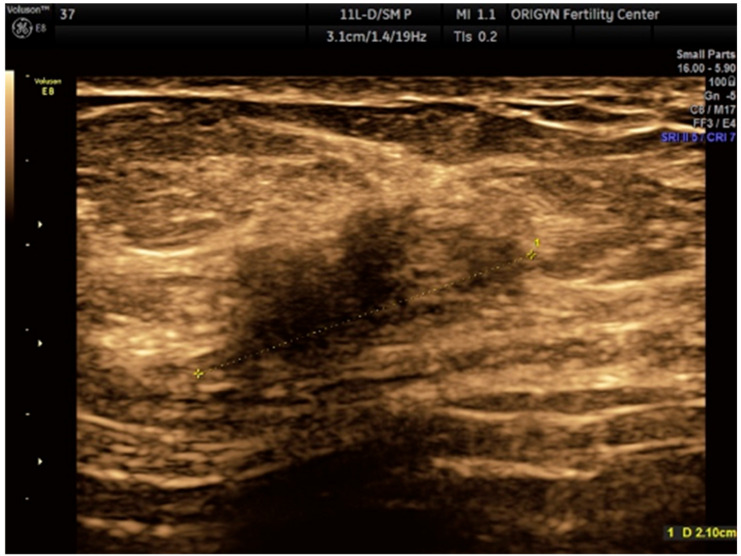
Ultrasound image hypoechoic solid mass with a polylobulated aspect and ill-defined margins.

The ultrasound revealed an irregular, heterogeneous, hypoechoic, oblong solid mass with ill-defined margins, located within the subcutaneous fat, with some infiltration in the abdominal muscle but no mobility to the deeper anatomical planes (Figure 1).

**Figure 2:**
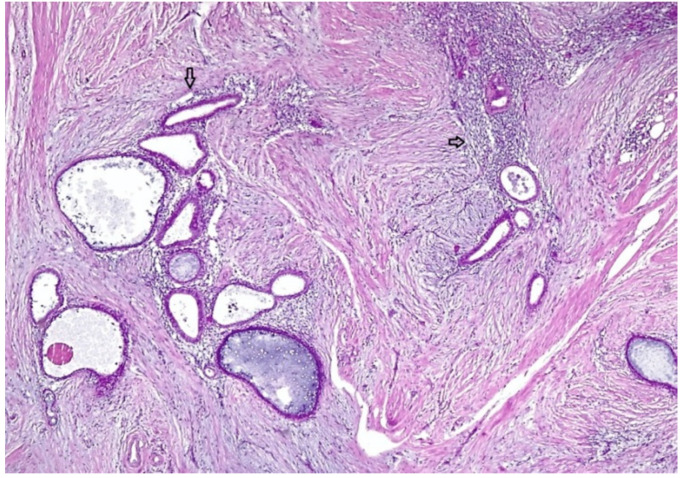
A histological aspect of the mass showing endometrial glands and stroma (arrows) included within conjunctive tissue.

The excised specimen in both cases was microscopically described as the following: fragment of connective-muscle-fat tissue with glandular components and endometrial-like stroma, with intra- and extracellular hemosiderin deposits, lymphoplasmacytic infiltrate, and fibrosis. Therefore, the presumed diagnosis of parietal endometriosis was histologically confirmed (Figure 2).

### 2.2. Case 2

**Figure 3:**
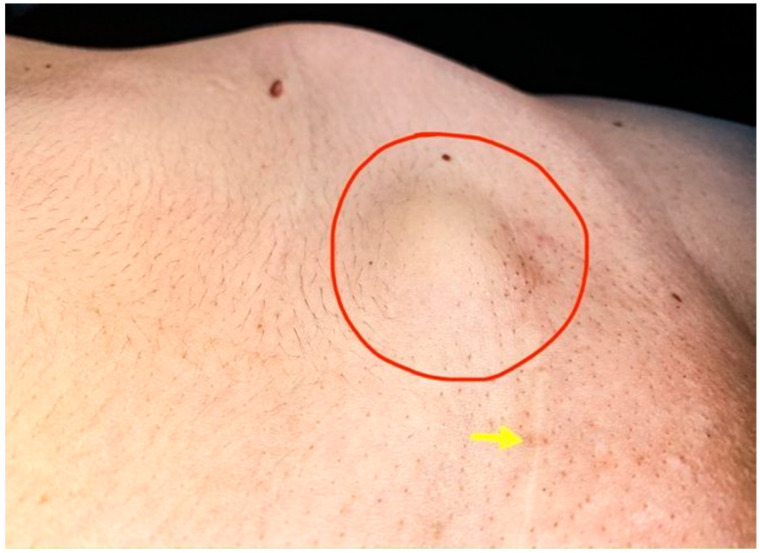
At the level of Pfannestiel scar (yellow arrow) a prominent painful mass around 3 cm (red circle) without mobility to the deep anatomical planes.

A 36-year-old Caucasian woman, G1P1 (one birth by caesarean section two years prior, in 2016), presented complaining of cyclic pain on the C-section scar, as well as of moderate to severe dysmenorrhoea and dyspareunia, two symptoms well known to be associated with endometriosis. She also pointed to a painful, palpable, small firm mass of ~3 cm in the lower abdomen wall, at the site of the caesarean section scar (Figure 3).

This patient had a history of ovarian endometrioma of ~6 cm removed by laparoscopy in 2014. Similar to the previous case, she was thrombophilic and had been administered Enoxaparin during pregnancy. Otherwise, her family history and her caesarean section were unremarkable, and there had been no natural births. She, too, was a non-smoker and a non-drinker and was not on any medication. 

**Figure 4:**
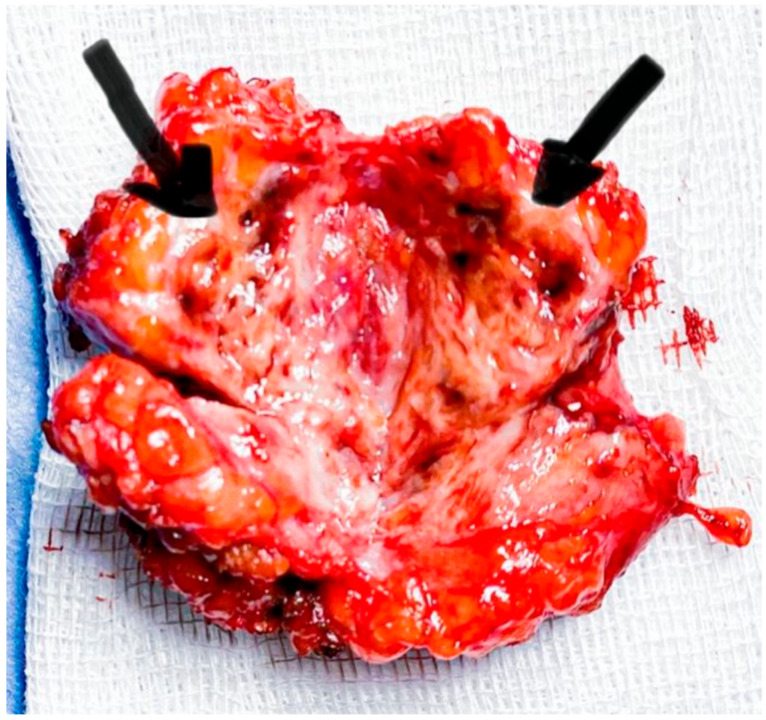
Sectioning revealed a fibrous tissue with numerous cysts with chocolate-like liquid (black arrows).

**Figure 5:**
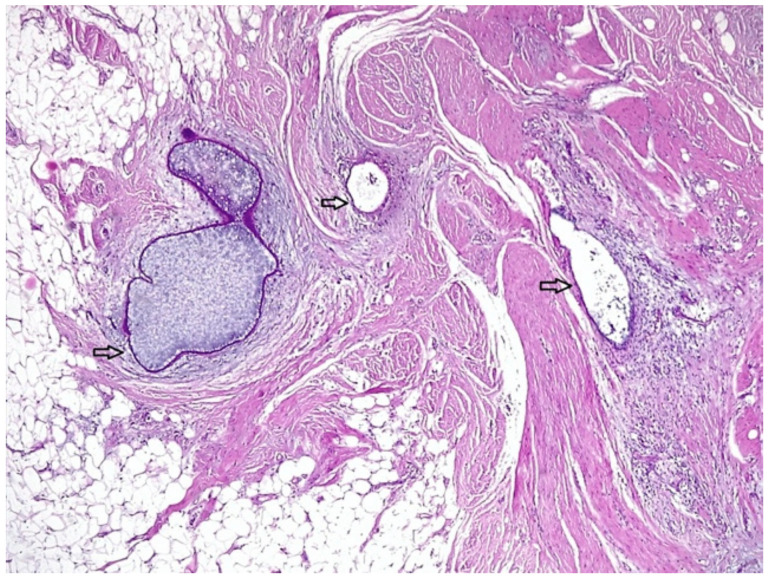
A histological aspect of the mass showing scarce endometrial glands and stroma (arrows) embedded within conjunctive and adipose tissue.

Upon the endovaginal ultrasound examination of the posterior wall of the uterus, we found a nodular, 25/26 mm mass with imprecise margins, diffuse Doppler signal, intramyometrial varicose veins, sliding sign absent posteriorly and present anteriorly. The right ovary, sized 35 mm/24 mm/13 mm, was adherent to the posterolateral wall of the uterus and sensitive upon mobilization with the probe. The left ovary, sized 32 mm/16 mm/10 mm, was also adherent to the posterior wall of the uterus and the left uterosacral ligament, and there was a cystic mass measuring 13/14 mm, suggestive of ovarian endometrioma. There was no sign of free fluid in the Douglas pouch. Transabdominal ultrasound revealed a heterogeneous, hypoechoic solid mass with a polylobulated aspect and ill-defined margins, located in the subcutaneous fat near the C-section scar. The mass had some scattered internal echoes and absent Doppler signal. Surgical excision was difficult due to invasion and lack of cleavage plans. The nodule section revealed a typical aspect (Figure 4), and the histologic exam confirmed the diagnosis (Figure 5).

## 3. Discussion

Scar endometriosis is an entirely iatrogenic disease with an incidence of 0.03–1% in women with a history of caesarean births [11]. It can also occur following gynecological surgeries (invasive or laparoscopic) if the uterine cavity is involved. It is very difficult to predict the time between the surgery and the onset of the disease. Data from case reports show that the time frame can range from 6 months up to 10 years [12].

The diagnosis is usually delayed because the signs and symptoms are not specific, and cyclic abdominal pain is not a pathognomonic sign for scar endometriosis. The differential diagnosis should be made with granuloma, desmoid tumor, lipoma, abscesses, sebaceous cysts, ventral hernias, or metastasis [7]. In our cases, the diagnosis was delayed around 2–3 years even if in the first case the pain has appeared 3 months after C-section. In our case, the patient considered that the pain was due to the surgery itself, and this led to a delayed diagnosis. The variable time between surgery and symptoms, the fact that this disease is rare, and similarities with other diseases determine a delayed diagnosis. It is still unknown what makes some forms aggressive with deep invasion into the abdominal wall, while most cases are limited up to abdominal aponeurosis because a direct link between surgery and diagnosis is not seen.

In addition to the anamnesis and clinical examination, the ultrasound should be the first-line imagistic method for diagnosis. High-frequency linear transducers are best for such assessment [13].

Upon ultrasound, a round or oval lesion with imprecise borders can be found at the site of the scar. The lesion typically appears as a hypoechoic area surrounded by a hyperechoic ring, with low or even absent blood flow when viewed in Doppler mode.

The treatment of scar endometriosis consists of wide lesion excision with a minimum 1 cm safe margin in order to avoid recurrence. In cases of incomplete excision of the lesion, the risk of recurrence varies between 12.5–28.6% [14]. In our cases, ultrasound evaluation 3 months after surgery shows no signs of residual or recidivant lesions. The interval dedicated to monitoring the patient after surgery to investigate whether there are outstanding or recurrent reminiscences is not yet clearly defined. The best way to prevent scar endometriosis is to avoid contamination with endometrial cells.

Scar endometriosis is, unfortunately, a disease with increasing incidence due to the rising rate of births by caesarean section and delays in diagnosis leading to abdominal wall invasion and serious health risks for the patient. It is very important to consider such a diagnosis in all patients of reproductive age who present with painful masses at the level of their scars. Among the various complications, delayed diagnosis can cause the patient to have to undergo complex and invasive surgical procedures, which may significantly impact their quality of life. The ultrasound is an accessible imagistic method and should be used routinely to evaluate any scar-related masses. Once a first nodule is detected, the entire scar must be assessed, and an ultrasound evaluation for ovarian endometriosis and deep endometriosis should be performed [4]. 

In what concerns the management strategies, this topic remains controversial. Because of the relatively low incidence of abdominal wall endometriosis, there is a relatively small body of evidence in the current literature. There are situations when surgery can be avoided, especially in cases of endometriomas or recurrent endometriomas that can be medically managed. Surgical intervention becomes imperative for patients that report perpetual pain, structures that are possibly malignant, infertility, or gradual increases in size.

Discrepancies regarding the usage of oral medication have been noted. They improve the overall health condition, but the success rate is low, and the recurrence chances once the treatment is ceased are high [15]. In contrast to oral contraceptives, danazol, progesterone, and gonadotropin-releasing hormone (GnRH), leuprolide is efficient during the first year, particularly for patients close to menopause. Unfortunately, it is associated with long-term repercussions and further correlated with adverse effects and does not reduce the size of lesions [16]. Therapy with oral contraceptives, progestins, medroxyprogesterone acetate, and GnRH agonists has been tried with minimal success. In some patients, the effects can be relatively long-lasting, but complete and long term regression of endometriosis is rare with hormonal therapy.

Among all the above mentioned, GnRH is also are widely used and dedicated to reducing pain and slowing the progression of endometriosis. In this way, GnRH’s pharmacokinetics led to conflicting results. While some authors argue that GnRH has no beneficial effect [17,18], we identified one study in which the size of lesions decreased after 6 months of use [19]. Aromatase inhibitors, GnRH agonists, and danazol are also dedicated agents, but there are insignificant data regarding the utility of these alternatives in strategies attributed to endometrioma therapy [20,21].

The role of danazol has been recently explored from various perspectives. To make a parallel and considering that it is well absorbed, this synthetic steroid causes gastrointestinal deficiencies in patients and a clinical panel identical to that observed in SARS-CoV-2-infected patients. Specific phenotypes that reflect metabolic and psychiatric disturbances have also been documented quite frequently. In this context, the limited number of studies carried out is explained, many of which were carried out before 2000 [22].

Through the prism of all data available, dienogest is a novel adjuvant to surgery and possesses an effective and tolerable compound that enjoyed a sudden emergence in recent years. Retrospectively, Bedaiwy et al. [23] summarized in their narrative review all existing information regarding the role of dienogest. From eighteen studies, the authors concluded individually that dienogest is useful not only for preventing post-surgical recurrence, but also to reduce the associated symptomatology.

To deepen this discussion, it seems that oral contraceptives were successfully used to lower the prevalence of endometrioma at the initial laparoscopy [24]. Having as support these initial observations, it was hypothesized that the formation of endometrioma can be bypassed by suppression [25,26]. Intriguingly, recurrence could be viewed as an integrated phenomenon in approximately 30% of the cases [27]. To strengthen this argument, we also identify three other studies that support the beneficial role of suppression [28,29,30].

Moreover, the risk is up to four times diminished following cystectomy, and women are prescribed twenty-four to seventy-two weeks of combined oral contraceptives (COCs) versus no treatment [30]. Additionally, in a recent systematic review and meta-analysis, the potent results obtained after the use of GnRH were further discussed [27], whereas those regarding levonorgestrel-releasing intrauterine device placement were inferior compared to ovarian suppression [31,32,33].

Cumulatively, COCs are the most eligible alternative for reducing large endometriomas [34], with not even norethindrone acetate (NEA) being totally risk-free despite its low costs and approval from the Food and Drug Administration (FDA). Nevertheless, regression of cysts in the first twelve weeks of treatment has been recently shown [35].

We identified only one study in which endometriosis was treated with ultrasound-guided ethanol injection. Bozkurt et al. [36] applied this technique to a 25-year-old woman with two previous caesarean sections diagnosed with a 3-cm abdominal wall endometrioma in the rectus muscle. The authors performed a Magnetic Resonance Imaging (MRI) and an ultrasound-guided needle aspiration through which was identified endometrial glands and stroma. The protocol they followed is as follows: (I) an injection of 1 mL of 95% ethanol; (II) administration for twelve weeks of oral contraceptives; (III) twelve weeks follow-up.

On the other hand, surgery remains the method of choice for clinicians since it offers two viable options; treatment and definitive diagnosis. It is crucial to first remove the nodule(s) and the adjacent fascia thoroughly. Unfortunately, the risk of novel lesions or reoccurrence of abdominal wall endometriosis is relative [15]. Some recommended strategies are using separate needles for the uterine and the abdominal closure, thorough washing and cleaning of the peritoneal cavity, and closing the uterine incision with care and without suturing the endometrium.

Sclerotherapy by ethanol injection before surgical resection represents another option, but intralesional ethanol injection may result in difficult-to-repair necrosis on the anterior muscles of the abdominal wall in large lesions. In endometriosis foci extending into the intraperitoneal region, it may also cause complications including chemical peritonitis and severe pain as a result of alcohol penetration into the peritoneum. In such patients, therefore, injections may be given in several sessions instead of a single session. Compared with the complications of surgical excision, the complications of sclerotherapy by ethanol are at a more acceptable level. Along with ethanol sclerotherapy, plasma energy is also attributed to such interventions, being less invasive, and optimal for women desiring to conceive [37].

Distinct surgical approaches may include polypropylene-mesh-closing, reserved for the cases with massive wall defect due to the invasion of the aponeurosis, a technique meant to lessen tissue tension. In wide surgical resections, complications including foreign substance reactions, mesh migration, and eventual incidence of hernia may appear due to the propylene mesh used. In the literature, abdominoplasty with polypropylene mesh is recommended for abdominal wall reconstruction in large lesions to reduce hernia development [37].

Although ultrasound is the most cost-efficient method of imaging evaluation, it is highly recommended and preferable that, in the case of abdominal wall endometriosis, further more detailed imaging investigations be conducted, such as CT/MRI. Such exams can offer important aspects regarding those lesions such as the extent of the tissue involvement and the state of all of the structures that come in close contact with the lesion.

There has been recent discussion about the usefulness of CT in the diagnosis of endometriosis. CT does not visualize pelvic organs but rather can be used to detect ureteral involvement and/or renal insufficiency [38].

However, the experience of the radiologist is essential considering that the average acquisition time for both high- and low-resolution MRI was twenty-four minutes. The values of specificity, sensitivity, and positive and negative predictive values (NPV/PPV) did not differ significantly, which is why both are considered valuable tools for detecting deep endometriosis extension [39]. Bermot et al. [40] investigate the detection performance of MRI of anterior pelvic endometriotic lesions, and while the two radiologists had an identical sensitivity (89.5%), the specificity value was as follows: 100% for the junior and 89.5% for the senior.

One good example is represented by the study of Burkett et al. [41], in which they aimed to quantify the value of pre-operative MRI in the management of women suffering from endometriosis. Out of one hundred and thirty-six patients, the associated methodology needs to be changed in 18.4% of the cases (*n* = 25). Whereas major changes were made in 8.1% (*n* = 11), minor changes were necessary in 13.2% (*n* = 18) of patients. Yap et al. [42] combined in their manuscript 98 MRI studies, from which 76 identified deep infiltrating endometriosis and 22 were normal. According to their results, 65 patients did not undergo any record, whereas the remaining subgroup underwent laparoscopy, operative and/or pathology reports (*n* = 37). With 195 days average time interval, middle compartment sensitivity (79.4%/specificity of 95.1%), posterior compartment sensitivity (76.5%/specificity of 99.4%) and the overall sensitivity and specificity of detecting bowel endometriosis of 94.4%, and 94.7% respectively, system benchmark diagnostic performance can be achieved through 3 T MRI.

Another retrospective study conducted by Bazot et al. [43], in which 666 patients were enrolled between 2005 and 2009, the overall prevalence of deep infiltrating endometriosis was 91.6% (*n* = 76 out of 83). In the present case, the sensitivity, specificity, NPV and PPV were 83.3%, 98.6%, 90.9% and 97.2%, respectively. Despite these data, it was recently demonstrated that laparoscopy is superior to MRI (*p* < 0.0001) especially in diagnosis chronic pelvic pain, but the MRI agreement with histopathology or laparoscopy was poor (*p* < 0.0455). The second diagnostic criterion was significantly improved in 96.9% of the cases (*p* < 0.0000) [44].

To date, no evidence leans towards or against the suture of the peritoneum concerning the incidence of AWE, nor is the change of gloves during the surgery recommended when completing the hysterorrhaphy. For abdominal wall endometriosis, total surgical excision is considered to be the gold standard for both diagnosis and treatment [45].

Although there is numerous evidence that the technique for uterine closure can be crucial for uterine scar healing, strong evidence regarding optimal techniques is little and there currently exist no national or international guidelines that obstetricians and gynecologists can rely on. Most randomized trials that have evaluated the uterine closure technique during caesarean have focused on the short-term operative complications without evaluating the impact on future pregnancies or the risk of developing caesarean scar endometriosis. Since there is no consensus on the matter, it is up to the surgeon and his clinical experience to adapt the techniques given the intraoperative findings [46].

## 4. Conclusions

The gold standard treatment in such cases is surgical, consisting of the excision of the mass within safe margins. This also allows for the specimen to be histopathologically analyzed to confirm the diagnosis. Moreover, an interesting approach would be finding an explanation regarding why some women are more susceptible to developing this condition while others are not and thereby determining if there are other factors involved such as genetics and environmental ones.
